# Human Sarcoma Growth Is Sensitive to Small-Molecule Mediated AXIN Stabilization

**DOI:** 10.1371/journal.pone.0097847

**Published:** 2014-05-19

**Authors:** Alessandra De Robertis, Federica Mennillo, Marco Rossi, Silvia Valensin, Patrizia Tunici, Elisa Mori, Nicola Caradonna, Maurizio Varrone, Massimiliano Salerno

**Affiliations:** 1 Molecular Oncology Unit, Siena Biotech Medicine Research Centre, Siena, Italy; 2 Department of Pharmacology, Siena Biotech Medicine Research Centre, Siena, Italy; 3 In Vivo Pharmacology Unit, Siena Biotech Medicine Research Centre, Siena, Italy; 4 Data Analysis Unit, Siena Biotech Medicine Research Centre, Siena, Italy; 5 MET Profiling Unit, Siena Biotech Medicine Research Centre, Siena, Italy; 6 Department of Medicinal Chemistry, Siena Biotech Medicine Research Centre, Siena, Italy; University of Washington, United States of America

## Abstract

Sarcomas are mesenchymal tumors showing high molecular heterogeneity, reflected at the histological level by the existence of more than fifty different subtypes. Genetic and epigenetic evidences link aberrant activation of the Wnt signaling to growth and progression of human sarcomas. This phenomenon, mainly accomplished by autocrine loop activity, is sustained by gene amplification, over-expression of Wnt ligands and co-receptors or epigenetic silencing of endogenous Wnt antagonists. We previously showed that pharmacological inhibition of Wnt signaling mediated by Axin stabilization produced *in vitro* and *in vivo* antitumor activity in glioblastoma tumors. Here, we report that targeting different sarcoma cell lines with the Wnt inhibitor/Axin stabilizer SEN461 produces a less transformed phenotype, as supported by modulation of anchorage-independent growth *in vitro*. At the molecular level, SEN461 treatment enhanced the stability of the scaffold protein Axin1, a key negative regulator of the Wnt signaling with tumor suppressor function, resulting in downstream effects coherent with inhibition of canonical Wnt signaling. Genetic phenocopy of small molecule Axin stabilization, through Axin1 over-expression, coherently resulted in strong impairment of soft-agar growth. Importantly, sarcoma growth inhibition through pharmacological Axin stabilization was also observed in a xenograft model *in vivo* in female CD-1 nude mice. Our findings suggest the usefulness of Wnt inhibitors with Axin stabilization activity as a potentialyl clinical relevant strategy for certain types of sarcomas.

## Introduction

The Wnt canonical signaling pathway (β-catenin dependent), is an important signaling cascade in metazoans, with crucial involvement in cellular proliferation, differentiation and development [1], [2]. Deregulated Wnt signaling has been associated with a variety of human pathologies [3] affecting different cell types and tissues including several types of cancer, diseases of the central nervous system and of the bone. In this respect, Wnt is in fact considered a key pathway in controlling normal osteogenesis [4], [5]. Axin1, the limiting component of the β-catenin destruction complex, is a multi-domain scaffold phospho-protein with tumor suppressor function involved in the coordination and regulation of several signaling pathways (Wnt, TGFβ and p53) and in the post-translational control of c-Myc protein level [6]–[11]. Osteosarcoma and fibrosarcoma are mesenchymal lineage malignancies affecting bone and soft tissues respectively. These tumors are characterized by aggressive growth of the primary lesions as well as development of distant metastases, with the lung representing one of the most common sanctuary sites [12]–[16]. Cytogenetic, molecular and gene expression profiling data revealed that sarcomas are characterized by complex karyotypes thus complicating the identification of consistent molecular signatures relevant for the identification of tumor “drivers” [16]. Mortality rates are still high, approaching 50% in soft tissue sarcomas and approximately 30% to 40% in osteosarcomas [15]–[17]. Experimental evidence supporting an involvement of the canonical Wnt pathway in mesenchymal tumors has been provided by multiple studies [18]–[21] although the molecular targets of Wnt signaling in sarcoma cells are still largely unknown. Specifically, canonical Wnt pathway activation in osteosarcoma and in other soft tissue sarcomas (STS) has been described involving mutations and/or altered expression levels of key pathway regulators (autocrine activation) [18]–[21]. Additionally, Wnt signaling can also be switched-on via crosstalk with other signaling pathways, including the phosphoinositide 3-kinase (PI3K)/AKT/mTOR pathway, which is frequently showed to be activated in sarcomas [22]–[24]. Consistent with a role in these tumors, reduction of *in vitro* and *in vivo* tumor growth and metastasis in osteosarcoma and fibrosarcoma respectively [25], [26] was achieved through ectopic expression of negative secreted modulators of the canonical Wnt pathway, such as of Wnt inhibitory factor 1 (WIF1) and the secreted Frizzled-related protein 3 (sFRP3; [27], [28]). β-catenin protein was found in the cytoplasm and nuclei of primary osteosarcoma cells [29], while, Wnt reporter activity was shown to be higher in various osteosarcoma cell lines compared with osteoblastic cells in the absence of exogenous Wnt stimulation [30]. De-regulation of the Wnt pathway in these tumors was also confirmed through an extensive analysis of human sarcoma tumors and sarcoma cell lines showing up-regulation of the Wnt canonical signaling by autocrine mechanisms in 50% and 65% of the examined cases, respectively [20]. Small molecule inhibition of Wnt signaling (mediated by the tankyrase inhibitors XAV939 [31] and IWR1 [32]), resulting in reduction of tumorigenic potential was also recently demonstrated in a class of soft tissue sarcomas [21], namely the malignant peripheral nerve sheath tumors (MPNSTs). Moreover, the tankyrase inhibitor JW74, showed stabilization of the tankyrase-target Axin2, down-regulation of the nuclear fraction of β-catenin and reduced *in vitro* cell growth in osteosarcoma cell lines [33].

In this study, we demostrate that a recently reported small molecule inhibitor of the canonical Wnt pathway, SEN461 [34], results in Axin1 stabilization followed by decreased total β-catenin levels in the osteosarcoma cell lines. Using U2OS cells as a model, SEN461 treatment resulted in decreased Wnt transcriptional signaling activity, modulation of well reported Wnt target genes (*AXIN2* and *CDC25A*), Axin1 stabilization and increased amount of phosphorylated β-catenin associated with Axin1 within the destruction complex. As a consequence of the pharmacological treatment, we found reduction of oncogenic phenotype associated with the sarcoma cell lines as showed by anchorage-independent growth *in vitro*. In the fibrosarcoma cell line HT-1080, the acute stabilization of the Axin1 protein, sustained by SEN461 treatment, negatively impacts the expression of the proto-oncoprotein c-Myc, an important mediator of sarcoma growth, *in vitro* and *in vivo*. Our findings support the pharmacological stabilization of Axin1 as potential therapeutic treatment for specific subtypes of sarcoma tumors.

## Materials and Methods

### Cell Lines

The cell lines U2OS, 143B, G292, HT-1080 and HEK293 were obtained from the American Type Culture Collection (ATCC) and cultured according to the supplier’s recommendations. Mouse Wnt3a containing conditioned media (Wnt3a-CM), and control conditioned media (CTR-CM) from mouse L cells, were harvested according to ATCC protocol.

### Plasmids, Wnt Reporter Activity, and Lentiviral Vectors

The generation of TCF-Luciferase and TA-Renilla reporter plasmids as well as IC_50_ determination and calculation was reported previously [34]. Human AXIN1 and WNT3A cDNAs were purchased from Origene as “transfection ready” plasmids. Dominant negative TCF4 cDNA was purchased from Upstate. Luciferase reporter of the nucleolin promoter (pNucL14) and FLAG-c-Myc expressing vector were from B. Amati. Luciferase reporter of the Hes5 promoter and Notch1-IC expressing vector were from I. Screpanti. Lentiviral vectors for inducible dominant negative TCF4 (rLV-EF1-tTS, rLV-EF1-rtTA and rLV-TRE-CMV-HA-TCF4DN) were purchased from Vectalys.

### Immunoblotting, Immunofluorescence, Confocal Analysis and Antibodies

Total, nuclear and cytosolic cells lysates were prepared as described previously [31]. Commercial antibodies used in this study include anti-Axin1, anti-β-catenin, anti-P-β−catenin Ser33/Ser37/Thr41 and anti-HA from Cell Signaling Technologies, anti-p21 (Santa Cruz), anti-tankyrases (Abcam), anti-Tubulin and anti-c-Myc from Calbiochem, anti-GAPDH and anti-β-actin (Sigma). For co-localization experiments, U2OS cells were plated directly on coverslips coated with Fibronectin (Invitrogen). After transfections and incubation treatment of 24 h, cells were washed once with 1X PBS and then fixed with 4% paraformaldehyde for 15 min. After three washes in PBS, cells were permeabilized with 0.1% Triton X-100 for 10 min and washed three times with TBS (Bio-Rad). Cells were subsequently blocked with 3% BSA +1% normal goat serum (Invitrogen) for 30 min and incubated overnight at 4°C with anti-P-β−catenin Ser33/Ser37/Thr41 antibody. After 24 h, cells were washed three times with TBS and incubated for 1 h with the following secondary antibody Alexa Fluor 546-conjugated goat anti-rabbit IgG (Invitrogen, 1∶1000). Finally, coverslips were mounted onto glass slides using Prolong Gold Antifade reagent with DAPI (Invitrogen). Cells were examined under laser confocal fluorescence microscope with plan-apochromate 40x and 63x/1.4 oil-immersion objective (LSM 510 Meta, Axiovert 200, Zeiss). Co-localization experiments were performed in triplicate, and data/images presented are the average of at least 10 random fields for each independent experiment.

### Real-time Quantitative RT-PCR

RNA extraction and quantitative RT-PCR (qPCR) expression analysis was performed as described previously [34]. Gene expression analysis was performed using the human housekeeping gene RPL13A. Primers for the hAXIN2, hCDC25A, hc-MYC, hFZD4, hDVL2, hCSNK1G and hVEGF-A were the following: Fw: 5′-CAAGGGCCAGGTCACCAA-3′ Rv: 5′-CCCCCAACCCATCTTCGT-3′; Fw: 5′-CTCCTCCGAGTCAACAGATTCA-3′ Rv: 5′-CAGCCACGAGATACAGGTCTT-3′; Fw: 5′-GGCTCCTGGCAAAAGGTCA-3′ Rv: 5′-CTGCGTAGTTGTGCTGATGT-3′; Fw: 5′-GTCTTTCAGTCAAGAGACGCTG-3′ Rv: 5′-GTTGTGGTCGTTCTGTGGTG-3′; Fw: 5′-TCAGCAGCGTCACAGATTCC-3′ Rv: 5′-GTCTCCCCGCTCATTGCTC-3′; Fw: 5′-ATGGACCATCCTAGTAGGGAAAA-3′ Rv: 5′-CACATCCTATCTTCTTGCCAACC-3′; Fw: 5′-AGGGCAGAATCATCACGAAGT-3′ Rv: 5′-AGGGTCTCGATTGGATGGCA-3′.

### Transfections, Infections, and Reporter Assays

Transfections were carried out using Lipofectamine 2000 (Invitrogen) according to the manufacturer’s instructions. Lentiviral expression of TCF-Luc, TA-Renilla, and the inducible dominant negative TCF4, were carried out following Vectalys instructions. For reporter assays, luciferase activities were performed as described previously [34].

### Soft Agar, Scratch, and Tube Formation Assays

Soft agar assay and analysis was carried out as previously described [34]. For scratch assay, confluent monolayer of U2OS and HT-1080 cells were subjected to scratch using a sterile pipette tip. Wounded monolayers were washed to remove debris and incubated with SEN461 at different concentrations for 20 hours. After the incubation period, images were taken under an Axiovert 200M (Zeiss) microscope equipped with a CCD camera, and the gap width was measured using MetaMorph 6.3 software (Universal Imaging Corp.) to calculate the area covered by cells (pixels unit). The tube formation assay was performed using growth factor-reduced Matrigel from BD (BD Biosciences; San Jose, CA, USA). Matrigel was thawed overnight at 4°C and was diluted (1∶1) with phenol red-free DMEM without serum. 100 µl of the diluted matrigel was used to coat the wells of a 96 well plate and was allowed to polymerize for 2 hours at 37°C. After equilibrating the gel with the complete growth medium, 15×10^3^ cells were seeded in each well and incubated for 20 hours. The tube formation was monitored under a phase contrast microscope (Axiovert 200, Zeiss) and imaged using MetaMorph system (Universal Imaging Corp).

### Statistical Analysis

Data were analyzed using GraphPad Prism 5.03 (GraphPad Software Inc, La Jolla, USA) and Matlab 2011b. Statistical analysis for quantitative RT-PCR, co-localization, and soft agar assays was performed by the Student’s t test. Expression values of genes of interest were calculated based on ΔΔCt type of analysis normalizing on RPL13A reference gene. Randomization procedure considering the tumor mass has been in-house developed and implemented in Matlab. Subjects are sorted according to their measured tumor masses. At each step a number equal to the experimental groups is taken and randomly assigned to a group according to a random permutation of the groups index. In this way it is possible to create a subject randomization in which each group has the same number of subjects and similar statistics in terms on tumor mass mean and standard deviation of the original complete sample.

### Compounds

SEN461 and SEN973 were designed and synthesized at Siena Biotech as previously described [34]. XAV939 and Suramine were purchased from Maybridge and Sigma respectively.

### In vivo Animal Model and Pathway/targets Modulation at the Tumor Site

Female athymic nude mice (6 weeks old, Crl:CD-1 nude mice) were obtained from Charles River Italia (Calco, Italy). HT-1080 cells (6×10^∧^6 cells) were resuspended in 200 µl of PBS 1X and injected subcutaneously in the right flank of the mice. Body weight and tumor measurements (using a digital caliper) were performed twice a week. Randomization was performed on day 12 after cell injection by comparing tumor mass and treatments started on day 13 when tumors reached a median size of 246 mm^3^ (range = 230–268 mm^3^). SEN461 was formulated in 0.5% w/v Methocel in MilliQ water and administered PO by oral gavage twice a day at 30 mg/kg for seven consecutive days in a total of 15 animals (n = 5 for each time point). Control animals (n = 5) were administered with the vehicle for the same time period. On treatment day 19, the mice were euthanized under inhalant anesthesia with isoflurane and tumor and blood samples were collected at three different time points (1–4–8 h, n = 5 animals for each time point) after last treatment and subjected to gene expression analysis and compound quantification. All mice were maintained in a conventional-specific-pathogen-free facility according to the NIH guidelines using an approved Animal Care and Use Committee protocol. During all the experimental time period, mice were housed (5 animals/cage) in individually ventilated solid floor plastic cages (Sealsafe Plus GR500, Tecniplast-Gazzada (VA) – Italy) using as bedding the Sawdust SCOBIS UNO by MUCEDOLA S.r.l (via Galileo Galilei 4, 20019 Settimo Milanese, Milano, Italy). Cages were maintained at a temperature of 20–24°C and humidity of 40–70% (Siemens Desigo Apparatus ver. 4.1) with 70 air changes/hour in the cage (10–15 air changes/hour in the room). Animals were subjected to a 12-hour light/12-hour dark cycle and have free access to a standard pelleted commercial laboratory diet (Certified Rodent irradiated 4RF21– Mucedola, Italy) and to municipal tap water, purified by reverse osmosis and autoclaved. The study was conducted in compliance with Decreto Legislativo January 27, 1992, N. 116, Gazzetta Ufficiale N. 40 February 18, 1992, (Directive N. 86/309/CEE) concerning protection of animals used for scientific purposes. The project was authorized by the Italian Institute of Health (48/2009/B). The experimental protocol was rewieved and approved by the Internal Ethical Committee.

## Results

### Osteosarcoma Cell Line U2OS is Sensitive to Pharmacological and Genetic Wnt Modulation

**Figure 1:**
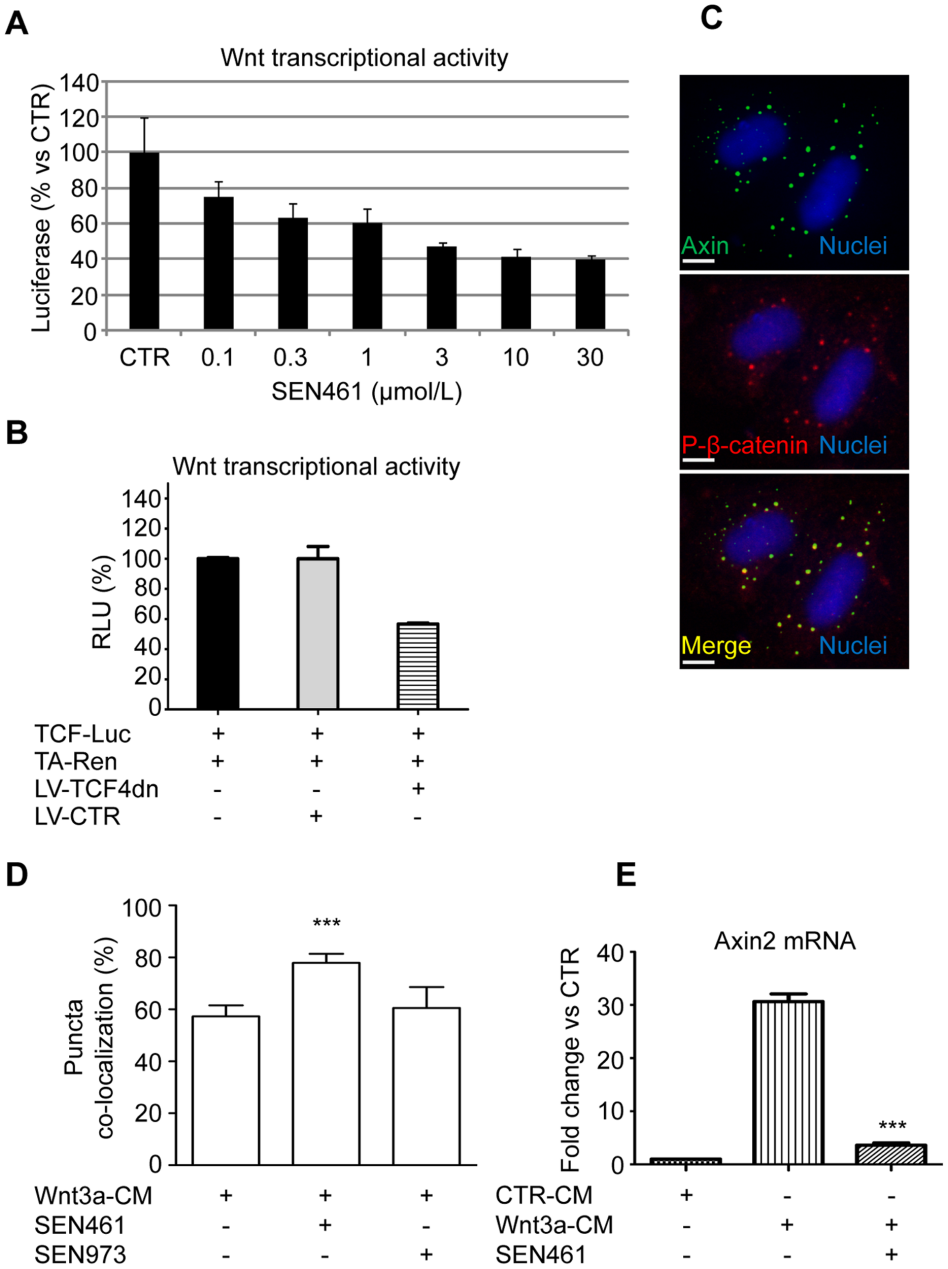
U2OS cells are sensitive to genetic and pharmacological Wnt pathway modulation. (**A**) U2OS cells infected with LVTCF-Luciferase and LVTA-Renilla were exposed to different amounts of SEN461 and the Wnt transcriptional activity was measured 24 h later. (**B**) The effect on Wnt transcriptional activity after inducible (10 ng/ml of doxycyclin) lentiviral infection with TCF4dn was measured by reporter activity. (**C**) Representative images for U2OS cells expressing Axin1-GFP (green) stained for P-β-catenin S33/S37/T41 (red) and their co-localization (yellow). Scale bar = 10 µm. U2OS cells, transiently transfected with GFP-tagged AXIN1 and stimulated with Wnt3a CM were either treated overnight with 10 µmol/L of SEN461 or with the inactive analogue SEN973 at the same concentration for the same length of time. (**D**) The bar-graph showed the result of three independent experiments where the number of Axin1/P-β-catenin co-localization puncta was counted in 5 different fields and normalized vs. untreated-Wnt3a-CM control. Data represent means ± SEM. ***, P<0.05 relative to control cells (Student t test). (**E**) Quantitative RT-PCR assay to measure the effect of SEN461 treatment after Wnt3a CM stimulation on the mRNA level of the Wnt target gene *AXIN2*. Data represent means ± SEM. ***, P<0.0001 relative to Wnt3a stimulated cells (Student t test).

The small molecule SEN461 inhibits canonical Wnt signaling pathway in cellular models, through an Axin stabilization mechanism [34]. To evaluate and characterize *in vitro* the potential activity of SEN461 in modulating Wnt signaling in a sarcoma background, we used the osteosarcoma cell line U2OS. These cells (free of mutations involving *APC*, *AXIN* and β*-catenin*, according to the Sanger Institute Database), were infected with TCF-Luciferase and TA-Renilla and incubated with different amounts of SEN461 for twenty-four hours. As showed in Figure 1A, the molecule reduced TCF-dependent reporter activity (expressed as the ratio of TCF-Luciferase/TA-Renilla activity) in a concentration dependent fashion (thus confirming the previous data obtained with the tankyrase inhibitors JW74 [33] and WIKI4 [35] in the U2OS cells). In the same cellular system, genetic down-modulation of the Wnt pathway, mediated by inducible lentiviral expression of dominant-negative TCF4 (LV-TCF4dn) inhibited endogenous TCF reporter activity (Figure 1B). To further characterize the mechanism of action of SEN461 with respect to specific Wnt pathway components, we evaluated the percentage of Axin1 protein co-localizing with phosphorylated β-catenin in the “destruction complex”, (a prerequisite for proteasome-mediated degradation of β-catenin). When U2OS cells, transiently transfected with GFP-tagged Axin1, were stimulated with Wnt3a exogenously provided in conditioned medium (Wnt3a-CM) containing SEN461, an increase in the amount of phosphorylated β-catenin associated with Axin1 was observed (Figure 1C and 1D). On the contrary, SEN973 [34], an inactive structural analog of SEN461 [34] did not produce any effect. Moreover, the mRNA levels for the Wnt/β-catenin target gene AXIN2, induced by Wnt3a CM, was inhibited by SEN461 treatment (Figure 1E). These results are consistent with the presence of an active Wnt signaling pathway in this cells [20], [26], [35], [36], and support the rationale for testing the pharmacological stabilization of Axin1, which might have clinical relevance for treatment of certain sarcoma sub-types. Conversely, we did not find any Wnt transcriptional modulation after SEN461 treatment in the fibrosarcoma line HT-1080 although exogenous expression of TCF4dn inhibited endogenous TCF reporter activity (data not shown). These data support previously results obtained in glioblastoma cells [34] and in the colorectal cancer cell line DLD1 (Figure S1), thus confirming that SEN461 is a bona fide an inhibitor of Wnt/β-catenin signaling.

### *In vitro* Phenotypic Consequences of SEN461 Treatment

**Figure 2:**
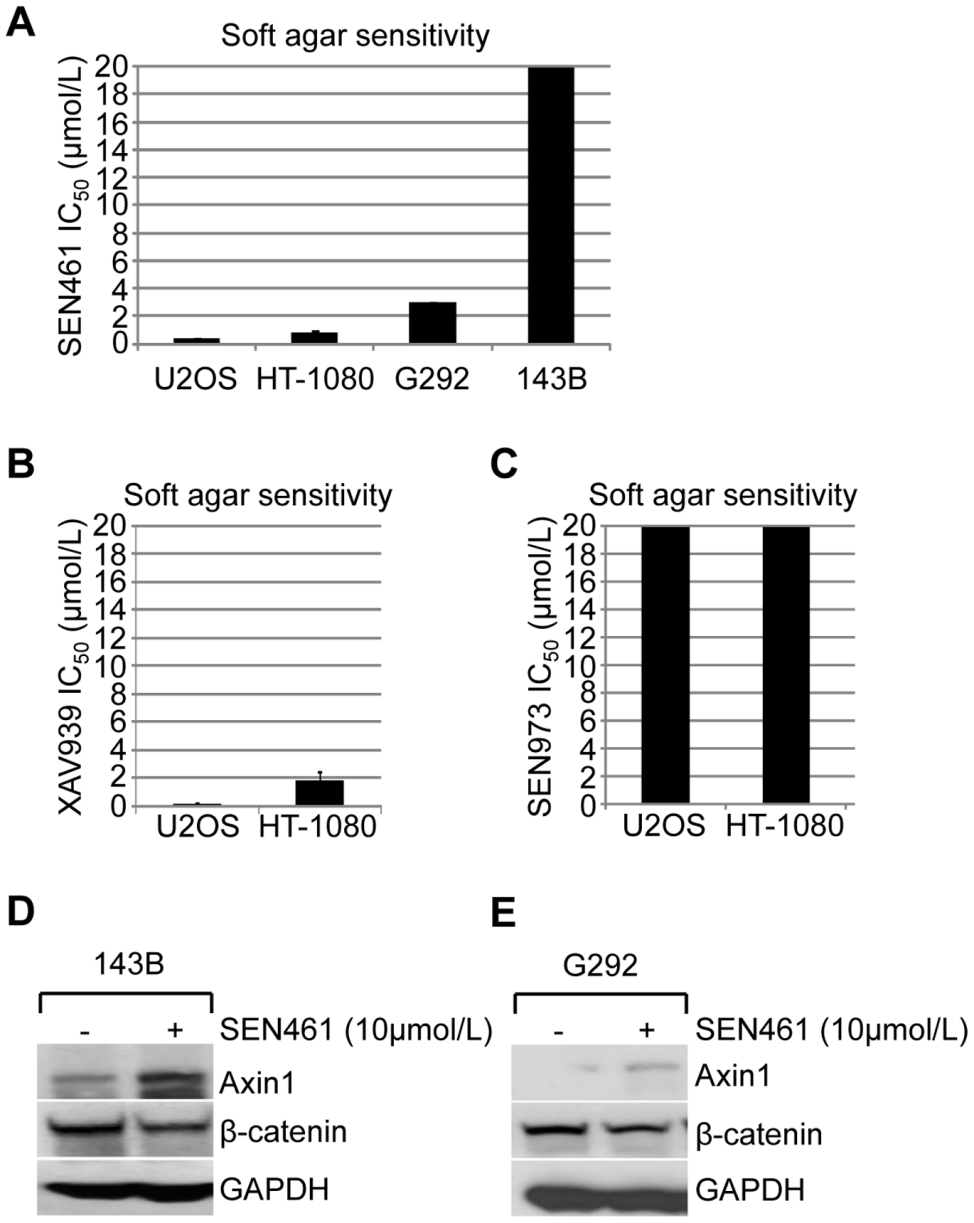
Effects of SEN461 on growth and on Wnt pathway components in sarcoma cells. (**A**) Summary of the half-maximal inhibitory concentration (IC_50_) for the four sarcoma lines examined in soft agar is shown, and ranked from lowest to highest. (**B, C**) IC_50_ for U2OS and HT-1080 cancer cells after XAV939 and SEN973 is shown, determined from the soft agar assay. Soft agar data (from two independent experiments) represent means ± SEM. (**D, E**) Western blotting analysis of Axin1 and β-catenin levels after SEN461 treatment in 143B and G292 osteosarcoma cells.

To explore some potential pharmacological effects of SEN461 on sarcoma cells, we examined its effects on anchorage-independent growth and cellular motility. Anchorage-independency and anoikis resistance, allow tumor cells to escape from the primary lesion and give rise to metastasis, a common feature of these tumor types [37]. As shown in Figure 2A, soft agar assay results demonstrated a strong inhibition by SEN461 of the ability to grow in anchorage-independent fashion for U2OS (osteosarcoma) and HT-1080 (fibrosarcoma) cell lines with IC_50_ values of 0.3 µM and 0.78 µM respectively. Comparable growth inhibition activity (IC_50_ values of 0.2 µM and 1.9 µM in U2OS and HT-1080 respectively) was also showed by the tankyrase inhibitor XAV939 [31] (Figure 2B). By contrast, no effect was shown by SEN973 (Figure 2C). Moreover, additional osteosarcoma cell lines have been tested (Figure 2A), showing a wide range of sensitivities to SEN461 treatment. The osteosarcoma cell line 143B, despite the lack of sensitivity (up to 30 µmol/L) to SEN461 treatment in the soft agar assay, showed Axin1 stabilization and down-regulation of total level of cytoplasmic β-catenin (Figure 2D), arguing that either the cell line is Wnt-independent or additional genetic/epigenetic alterations drive the growth and therefore consequently the insensitivity to SEN461. Axin stabilization associated to β-catenin down-modulation was also observed in the osteosarcoma cells G292 (Figure 2E). We then asked wheatear SEN461 treatment could have an impact on cellular motility. As showed in the Figure S2A neither U2OS nor HT-1080 cells showed inhibition of motility assessed by the two-dimensional scratch assay. The ability of HT-1080 cells to form aggressive angiogenic tumors in xenograft mouse models [38], their high level of secreted VEGF-A and the up-regulation of hypoxia-related genes observed in different human sarcoma tumors [39], led us to investigate the potential *in vitro* effects of SEN461 treatment on tubule-formation by the hypoxia-primed cells. As represented in the Figure S2B, tubule-formation activity measured after 24 hours of exposure to either SEN461 or the tankyrase inhibitor XAV939 [31] did not produce any change compared to DMSO treated HT-1080 cells. On the contrary, Suramin at a concentration of 3 µmol/L [40] induced a complete abrogation of tubule formation (data not shown). Moreover, SEN461 did not show any effect on tubule-formation activity, even on HUVEC cells (data not shown).

### SEN461 Effects at the Molecular Level

**Figure 3:**
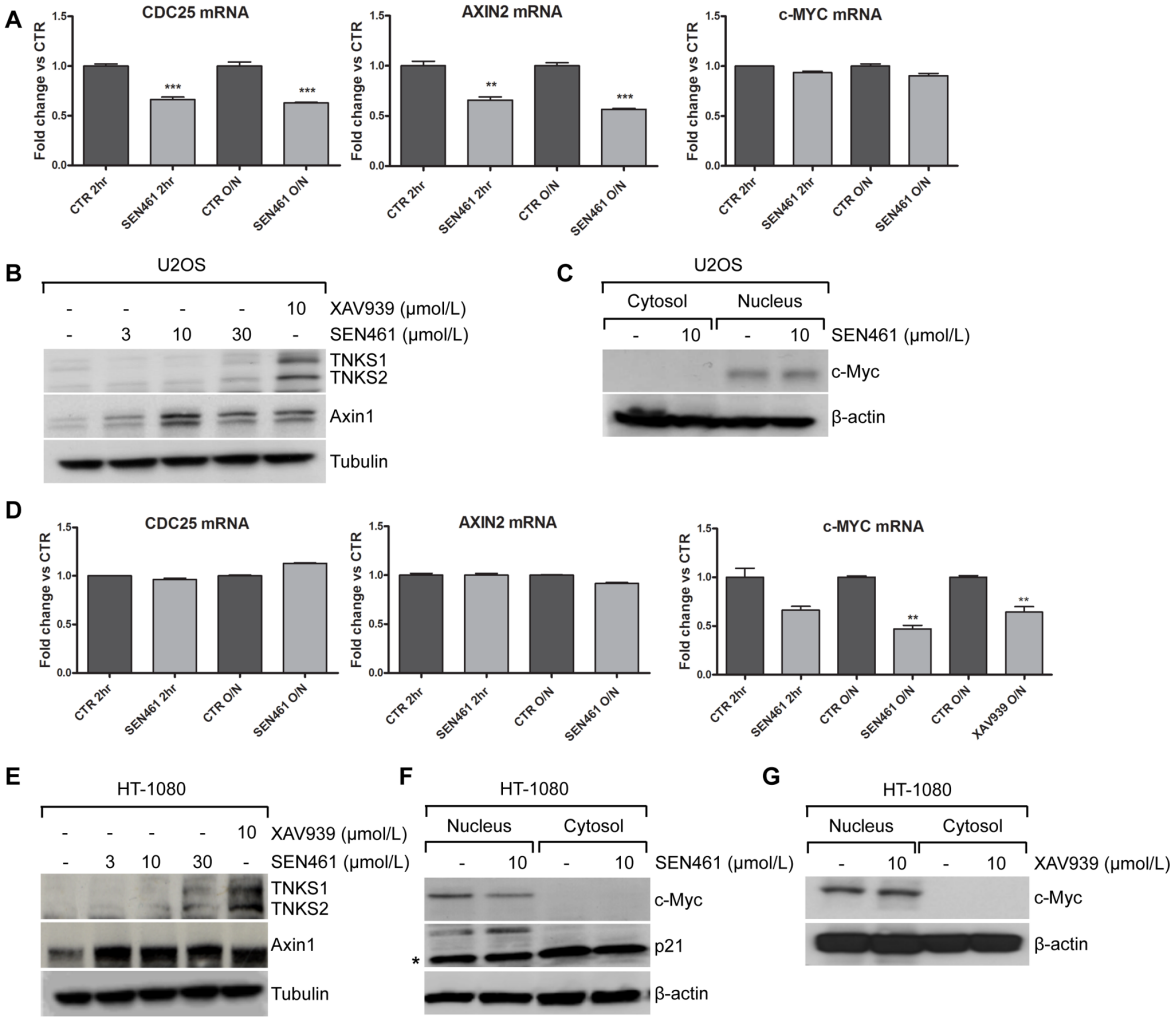
SEN461 effects on Wnt molecular components in sarcoma cells. (**A, D**) Effect of SEN461 or XAV939 on Wnt target genes mRNA expression measured by quantitative RT-PCR in U2OS and HT-1080 cells after 10 µmol/L treatment. Data (collected from three independent experiments) represent means ± SEM. **, P<0.05 ***, P<0.005 relative to control cells (Student t test). (**B, E**) Western blotting analysis of U2OS and HT-1080 cell lines treated with SEN461 or XAV939 overnight. Cytoplasmic cell lysates were probed with anti-Axin1, anti-TNKS1/2 and anti-Tubulin as loading control. (**C, F, G**) Western blotting analysis of U2OS and HT-1080 treated with SEN461 or XAV939 overnight. Cytoplasmic and nuclear cell lysates were then probed with anti-c-Myc, anti-p21 and anti-β-actin as loading control. The asterisk represents a background band migrating below the p21 band.

**Figure 4:**
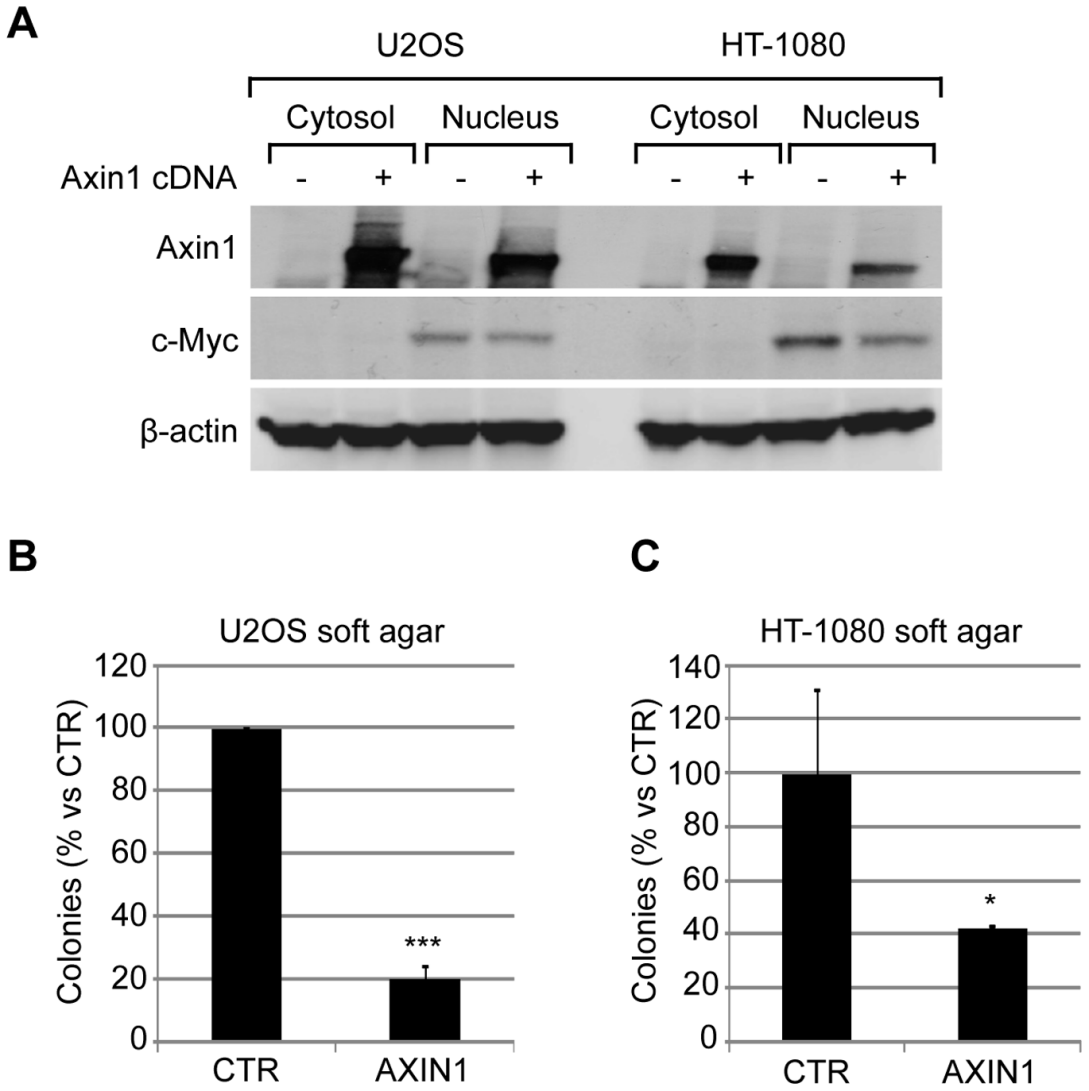
Axin1 over-expression phenocopies SEN461 activity at molecular and phenotypic level. (**A**) U2OS and HT-1080 cells were transiently transfected with AXIN1 cDNA. Cell lysates were then analyzed by Western blotting and probed with anti-Axin1, anti-c-Myc and anti-β-actin as loading control. (**B, C**) Ectopic expression of AXIN1 in U2OS and HT-1080 cells produced a strong reduction in their ability to grow in anchorage-independent fashion. Soft agar data (from two independent experiments) represent means ± SEM. *, P<0.05 ***, P<0.0005 relative to control cells (Student t test).

In order to link Axin1 stabilization, Wnt signaling and anchorage independent growth in sarcoma cells, we started to examine the effect of SEN461 treatment on key components of the canonical Wnt pathway. In U2OS cells, *AXIN2* and *CDC25A* mRNAs showed comparable down-regulation after either short or long exposure (two or twenty-four hours respectively) to 10 µmol/L of SEN461 (Figure 3A). Moreover, additional Wnt targets (*FZD4*, *DVL2* and *CSNK1G*) showed down-modulation at the mRNA level (Figure S3). On the contrary, the mRNA level of the Wnt target gene *c-MYC* was unaffected by overnight compound treatment in U2OS cells (Figure 3A) as well as in all the osteosarcoma lines tested in the soft agar assay (data not shown), arguing that it might not represent a direct transcriptional target of the Wnt signaling in the analyzed tumor cells. In fact, to further investigate this finding, stimulation of the canonical Wnt pathway in U2OS cells by Wnt3a conditioned medium led to an up-regulation of *AXIN2* and *CDC25A* mRNA without affecting *c-MYC* expression (data not shown), thus supporting our observation and confirming previous data [20]. At the protein level, SEN461 treatment induced concentration-dependent stabilization of Axin1 with a very slight stabilization of tankyrases (TNKS1 and TNKS2) only at the highest concentration of SEN461 (Figure 3B). No effect was noticed on c-Myc protein levels (Figure 3C). In the HT-1080 fibrosarcoma cells, among the Wnt target genes tested, c-MYC mRNA was the only one showing a significant down-modulation (Figure 3D) after overnight compound treatment at 10 µmol/L (comparable activity was also showed by XAV939). To evaluate the potential involvement of SEN461 in c-Myc transcriptional activity, we performed luciferase reporter assay on the nucleolar protein nucleolin [41], representing a genuine target of Myc mediated activation. Myc transcriptional activation was tested in HEK293 and HT-1080 cells transfected with the luciferase reporter plasmid pNucL14 [41] alone or in combination with FLAG-c-Myc expression plasmid. We found that SEN461 did not affect either Myc mediated activation or the basal activity of the reporter assay (Figure S4), arguing that SEN461 effect on c-Myc might occur at the post-transcriptional level. Additionaly, we also tested the potential inhibitory effect of SEN461 on Notch signaling in HEK293 cells transfected with the Hes5 promoter coupled to luciferase after stimulation with Notch1-IC expression plasmid (data not shown). Also in this case we did not detect any transcriptional effects. At the protein level, a robust concentration dependent stabilization of Axin1 induced by SEN461 exposure (Figure 3E) is coupled to a down-modulation of c-Myc followed by the up-regulation of the CDK inhibitor p21 (Figure 3F). Conversely XAV939 did not affect c-Myc protein level (Figure 3G). To examine whether exogenous expression of Axin1 affects the c-Myc/p21 axis, phenocopying the pharmacological effect produced by the small molecule, we transiently transfected HT-1080 cells with Axin1 expression vector and found indeed that to be the case (Figure 4A). On the contrary this was not observed in the U2OS cells (Figure 4A). C-Myc is a well-known key player in influencing and supporting the proliferative activity of many cancers [42], and p21, a direct target of Myc, was shown to be up-regulated upon down-regulation of c-Myc in different tumor types [43]–[45]. We then asked whether over-expression of Axin1 would affect the phenotypic behaviour of the two sarcoma cell lines examined more in depth. Axin1 over-expression exerted its known tumor suppressor function [46], [47], showing a profound effect on HT-1080 as well as on U2OS anchorage-independent growth ability (Figure 4B and 4C), phenocopying the pharmacological effects of SEN461 at the phenotypic level.

### SEN461 *in vivo* Activity

**Figure 5:**
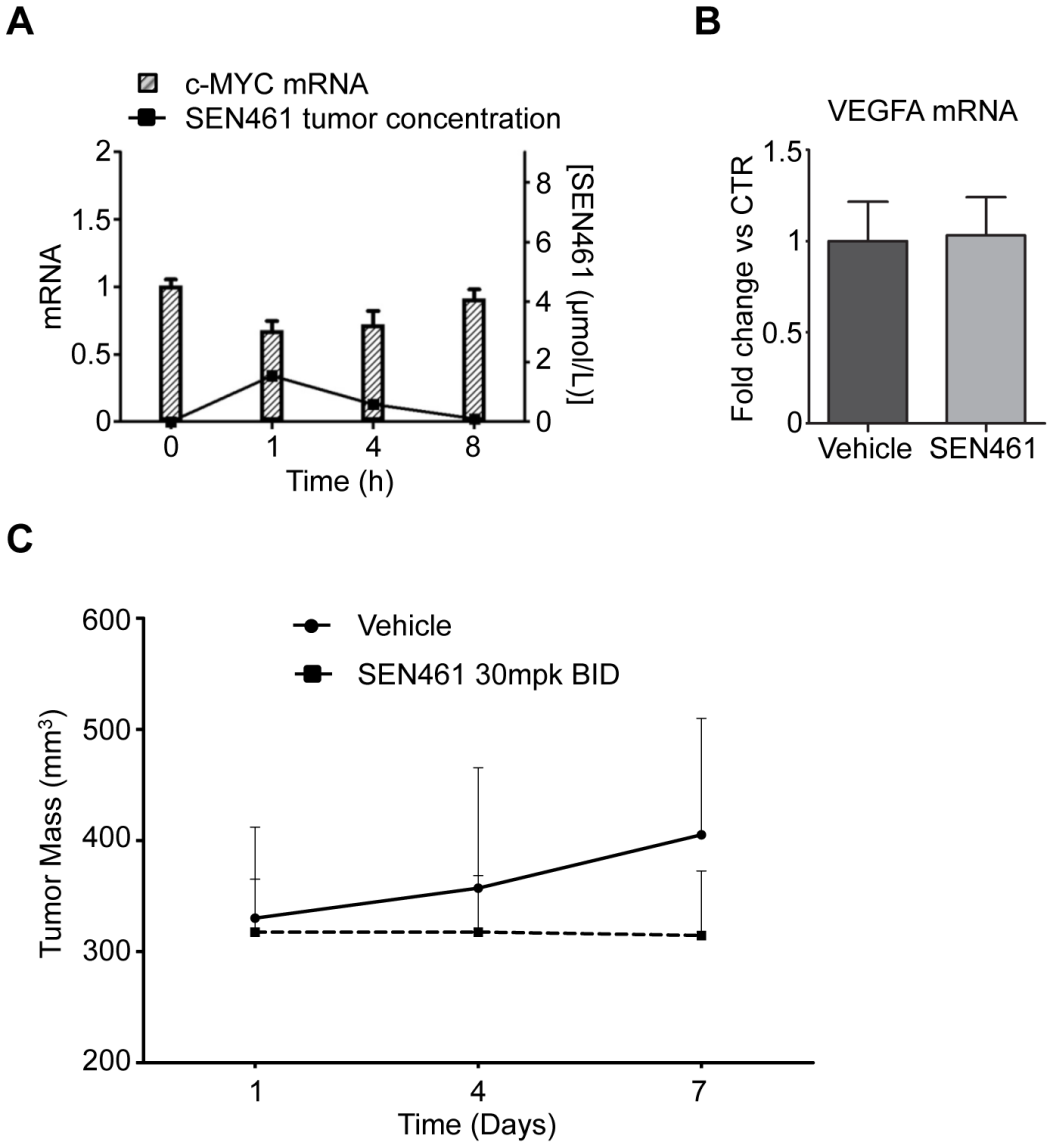
*In vivo* effects of SEN461 in HT-1080 xenograft model. (**A**) Pharmacokinetics and pharmacodynamics of SEN461 in mice. Concentration of SEN461 in tumors (black line) and relative *c-MYC* human mRNA values (columns) in HT-1080 xenograft tumors at 1, 4 and 8 hours after 30 mpk BID oral administration of SEN461. The data are presented as mean ± SEM (n = 5) and T0 represents *c-MYC* human mRNA value at 1 hour after vehicle administration in the control group. (**B**) mRNA level for the human *VEGFA* gene in HT-1080 xenograft tumors at 1 hour after 30 mpk BID of SEN461. The data are presented as means ± SEM (n = 5). (**C**) Antitumor activity of SEN461 in a HT-1080 xenograft tumor model. Treatment groups (5 mice per group) received 30 mg/kg twice a day for seven consecutive days.

Pharmacokinetic analyses showed that SEN461 administered orally (PO) at a dose of 30 mg/kg twice a day for seven days, yielded robust *in vivo* exposure with values of 6.6 µmol/L in the plasma and 1.5 µmol/L inside the tumor at 1 hour after the last dosing. The plasma and tumor concentration of SEN461 declined then to low nanomolar levels by 8 hours (Table 1). Analyses of mRNA extracted from HT-1080 xenograft tumors harvested at different time points after SEN461 administration revealed down-modulation of *c-MYC* compared to control animals (Figure 5A), without any significant effect on *AXIN2* or *CDC25A* (data not shown), in agreement with the *in vitro* data. As previously demonstrated in U2OS cells, *in vitro* activation of the canonical Wnt signaling pathway mediated by Wnt3a conditioned medium in HT-1080 cells led to an up-regulation of *AXIN2*, *SFRP1* and *NKD1* mRNA expression but not *c-MYC* (data not shown), indicating that also in these cells c-MYC might not represent a direct Wnt transcriptional target. To assess selectivity for the c-MYC primers, mRNA derived from mouse brain was tested in a qPCR assay, where no amplification was detected (data not shown). C-Myc is commonly found altered in primary sarcomas [48] and its depletion by shRNA inhibited *in vitro* and *in vivo* proliferation of HT-1080 and additional sarcoma cell lines [20], [49]. Moreover, analysis of mRNA levels for the *VEGF-A* gene in the HT-1080 derived xenograft tumors (Figure 5B), did not show any difference in the treated versus control animals; thus confirming the previous data and therefore excluding a direct involvement of SEN461 in interfering with angiogenic/neoangiogenic driven processes. Although the aim of the xenograft model was mainly focused on the analysis of potential pharmacodynamic biomarkers, SEN461 treatment at a dose of 30 mg/kg twice a day showed a tumor stasis effect on the tumor for the entire treatment period (Figure 5C). All animals receiving SEN461 twice a day for seven days, maintained their body weight with no significant changes (Figure S5A), correlating with absence of gross histological changes in the architecture of gastrointestinal tract (Figure S5B).

**Table 1:** Plasma and tumor exposure of SEN461 in mice.

Dose	Day	Time (h)	Cplasma (µmol/L)	Ctumor (µmol/L)	Ctumor/plasma
30mg/kg BID	7	1	6.62±2.08	1.54±0.35	0.23
30mg/kg BID	7	4	2.57±0.08	0.58±0.08	0.23
30mg/kg BID	7	8	0.57±0.19	0.10±0.02	0.17

Plasma an tumor levels of SEN461 in female Crl:CD-1 nude mide on day 7 after 30 mg/kg BID oral administration for seven consecutive days. C_tumor_ to C_plasma_ ratios did not vary with time. Plasma and tumor concentrations were reported as mean ± SD of 3 animals per time point, except for the tumor concentration at 1 h, which represents the mean of 2 animals.

## Discussion

Osteosarcoma and fibrosarcoma tumors are mesenchymal cancers sharing complex karyotypic changes with poorly defined recurring molecular events. Beside the use of systemic conventional chemotherapy, either in adjuvant or neo-adjuvant setting, there is an urgent need to find alternative therapies based on selected targets/pathways of the oncogenic cascades involved in local and systemic progression. We previously reported, that the Wnt inhibitor/Axin stabilizer SEN461, exhibited tumor growth inhibition activity in *in vitro* and *in vivo* models of glioblastoma. Here, we demonstrated that SEN461 reduces the tumorigenic potential of osteosarcoma and fibrosarcoma cell lines and confirm this activity is mediated largely through Axin stabilization. SEN461 affects Wnt transcriptional activity, influences the amount of β-catenin levels and modulates Wnt pathway components in the examined osteosarcoma cell lines. In U2OS cells, SEN461, was shown to modulate the canonical Wnt transcriptional target *AXIN2* toghether with *CDC25A*, recently described as an important mediator of Wnt-induced sarcoma cell proliferation both *in vivo* and *in vitro* [20]. A relevant role for Axin in reducing anchorage-independent growth was also phenotypically demonstrated in U2OS and HT-1080 cell lines by its overexpression. The *in vitro* and the *in vivo* data generated in the HT-1080 cell line, suggests that Axin mediated stabilization by SEN461 may also operate independently on the Wnt signaling pathway through either its tumor suppressor function or its scaffold activity for the c-Myc protein. In this respect, Axin1 was in fact demonstrated to be involved in the formation of a degradation complex for c-Myc [9], with its acute expression affecting c-Myc levels. Moreover, in the HT-1080 cell line, c-Myc protein turnover is highly deregulated compared to non-tumorigenic or to the U2OS osteosarcoma cells [50]. This makes c-Myc a relevant “driver” for tumorigenicity in the fibrosarcoma HT-1080 cells, as reported by the proliferation effect induced by gene knock-down [20], and explains the reduced tumorigenic potential evoked by SEN461 treatment. In fact, Myc modulation was also previously reported to be able to reverse the process of transformation even in tumors with high genomic complexity like sarcomas [51]. Pharmacological down-modulation of c-Myc protein level was also shown to be phenocopyed by Axin1 overexpression in HT-1080 but not in U2OS cells. Although the precise molecular target through which SEN461 exerts its anti-tumor activity has yet to be determined, similarities at the phenotypic level coupled with discrepancies at the molecular level (e.g. down regulation of c-Myc protein level) between XAV939 and SEN461 suggest that they act similarly but not identically. However, Axin involvement, either as a direct component of the Wnt signaling or indirectly seems to be relevant to reduce the tumorigenic potential of the sarcoma cells tested. In conclusion, the data presented here demonstrate pharmacological stabilization of Axin as a potential therapeutic opportunity to treat sarcomas.

## Supporting Information

Figure S1 **Wnt pathway modulation in DLD1 colorectal cancer cells after genetic manipulation or pharmacological treatment. (A)** Inhibition of Wnt transcriptional activity either by inducible (10 ng/ml of doxycyclin) lentiviral infection with TCF4dn or SEN461 treatment was measured by reporter assay. **(B)** Concentration dependent inhibition of Wnt transcriptional activity induced by SEN461 treatment in DLD1 cells transiently transfected with TCF-Luciferase and TA-Renilla. **(C)** Time dependent effect of SEN461 on *AXIN2* mRNA levels measured by quantitative RT-PCR. **(D)** Western blotting analysis of DLD1 cells treated with different amount of SEN461 overnight. Cytoplasmic cell lysates were then probed with anti-Axin1, anti-Axin2, anti-β-catenin, anti-P-β-catenin and anti-GAPDH as loading control.
(TIF)

Figure S2 **SEN461 phenotypic effects on sarcoma cells. (A)** Ability of SEN461 to affect cellular motility was examined by scratch assay (in two independent experiments) in U2OS and HT-1080 cells. **(B)** The effect of SEN461 on angiogenesis was examined by tube formation assay (in three independent experiments) in HT-1080 cells.
(TIF)

Figure S3 **SEN461 effects on Wnt molecular components in U2OS cells.** The effect of 10 µmol/L treatment with SEN461 on the mRNA levels of Wnt target genes *FZD4*, *DVL2* and *CSNK1G* was measured by quantitative RT-PCR. Data represent means ± SEM. **, P<0.05 ***, P<0.005 (Student t test).
(TIF)

Figure S4 **SEN461 doesn’t affect Myc transactivation of the nucleolin promoter. (A)** HEK293 and **(B)** HT-1080 cells were transiently transfected with the mouse nucleolin reporter plasmid pNucL14 and the FLAG-c-Myc expression vector either alone or in combination and then treated with DMSO or SEN461. Data (from two independent experiments), normalized by cotransfection of TA-Renilla luciferase represent means ± SEM.
(TIF)

Figure S5 **Effects of SEN461 on body weight and intestinal tissue. (A)** Average body weight graph and representative histological sections **(B)** of intestinal tissue from mouse treated with vehicle or SEN461 for 7 days stained with hematoxylin and eosin.
(TIF)
