# Supplementary figures and images for: Human Sarcoma Growth Is Sensitive to Small-Molecule Mediated AXIN Stabilization
Source: PLoS One. 2014 May 19;9(5):e97847. doi: 10.1371/journal.pone.0097847 (PMC4026528; doi:10.1371/journal.pone.0097847)

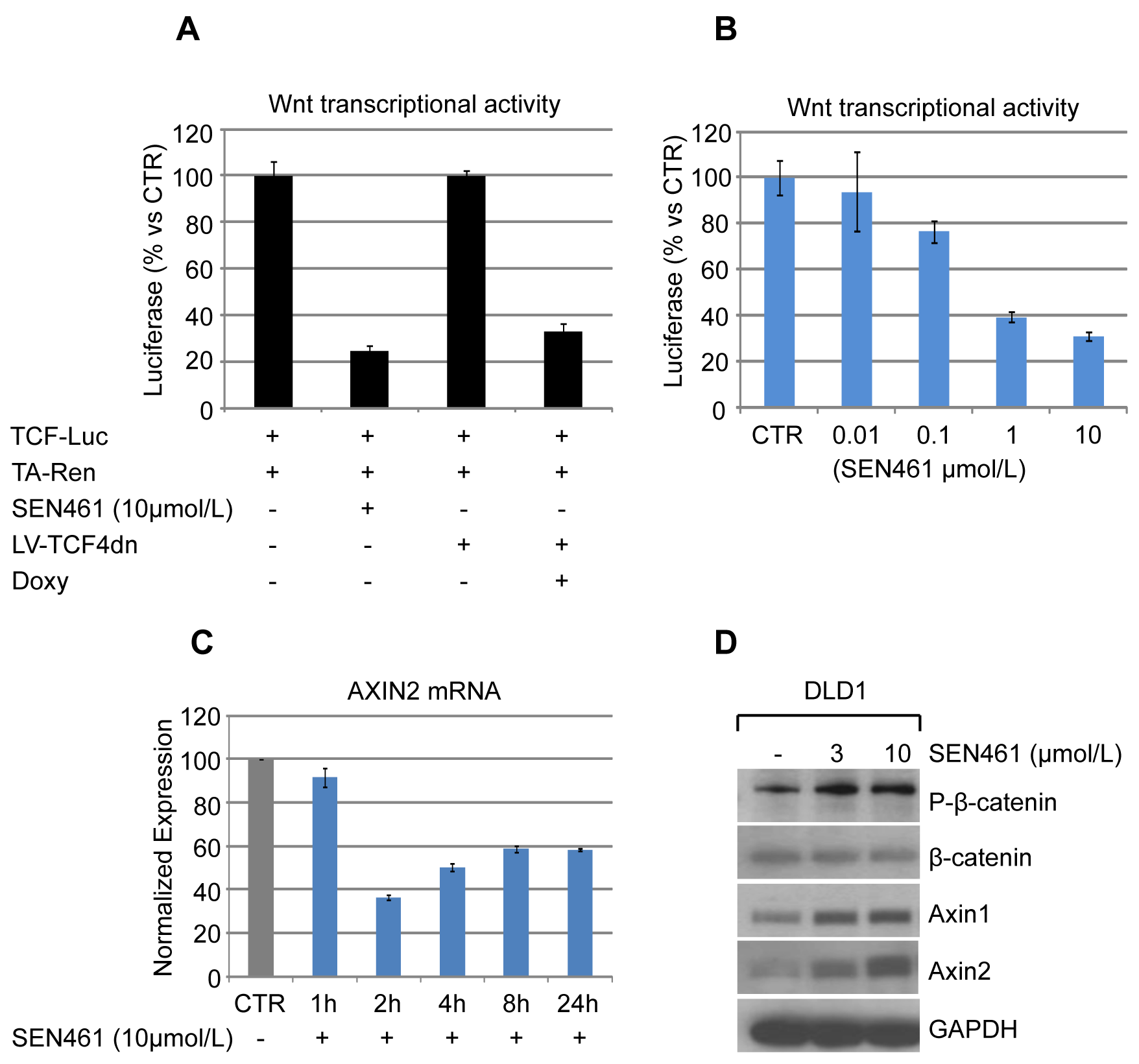

Supplement: Figure S1 — Wnt pathway modulation in DLD1 colorectal cancer cells after genetic manipulation or pharmacological treatment. (A) Inhibition of Wnt transcriptional activity either by inducible (10 ng/ml of doxycyclin) lentiviral infection with TCF4dn or SEN461 treatment was measured by reporter assay. (B) Concentration dependent inhibition of Wnt transcriptional activity induced by SEN461 treatment in DLD1 cells transiently transfected with TCF-Luciferase and TA-Renilla. (C) Time dependent effect of SEN461 on AXIN2 mRNA levels measured by quantitative RT-PCR. (D) Western blotting analysis of DLD1 cells treated with different amount of SEN461 overnight. Cytoplasmic cell lysates were then probed with anti-Axin1, anti-Axin2, anti-β-catenin, anti-P-β-catenin and anti-GAPDH as loading control. (TIF) [file pone.0097847.s001.tif]

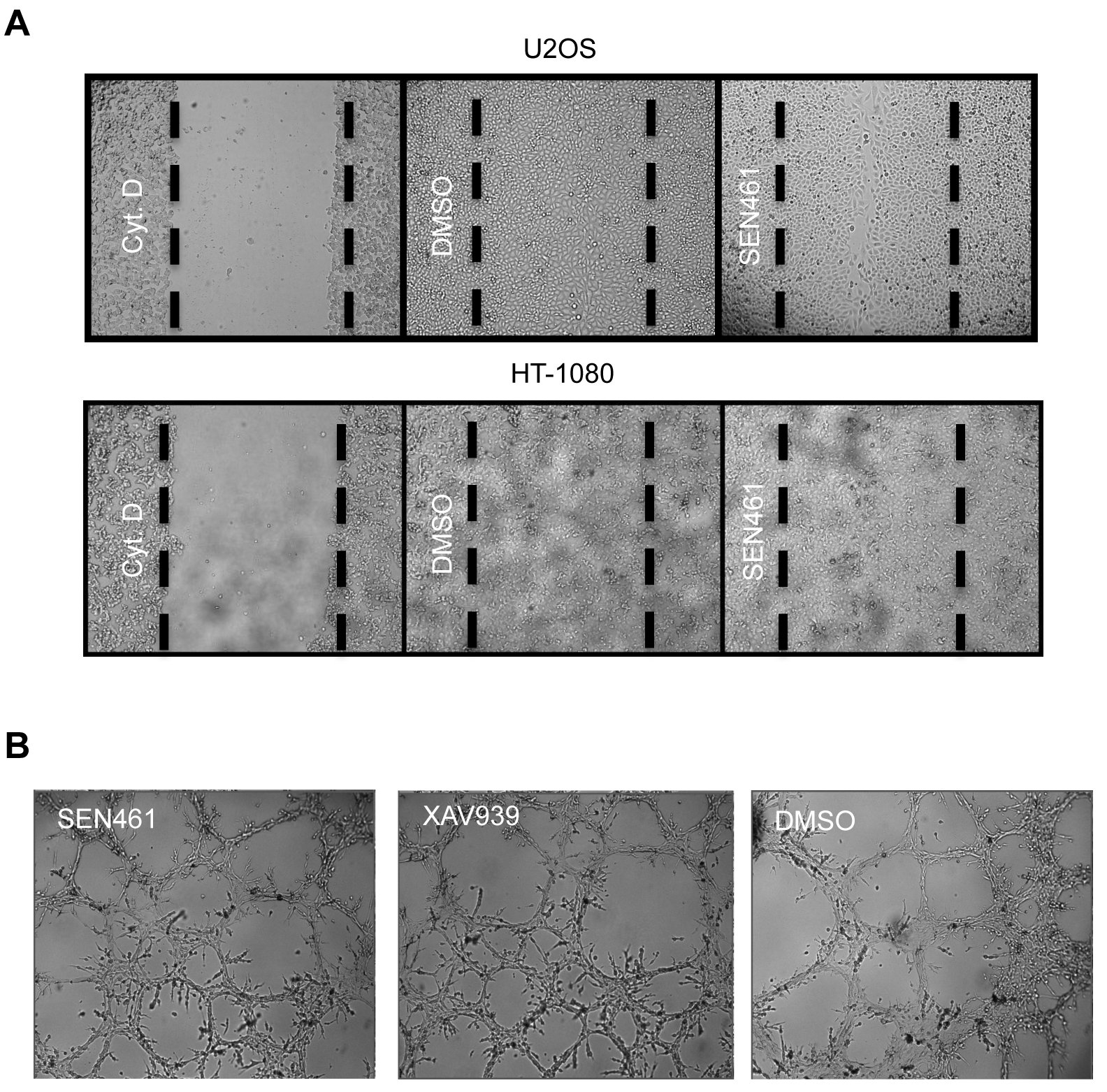

Supplement: Figure S2 — SEN461 phenotypic effects on sarcoma cells. (A) Ability of SEN461 to affect cellular motility was examined by scratch assay (in two independent experiments) in U2OS and HT-1080 cells. (B) The effect of SEN461 on angiogenesis was examined by tube formation assay (in three independent experiments) in HT-1080 cells. (TIF) [file pone.0097847.s002.tif]

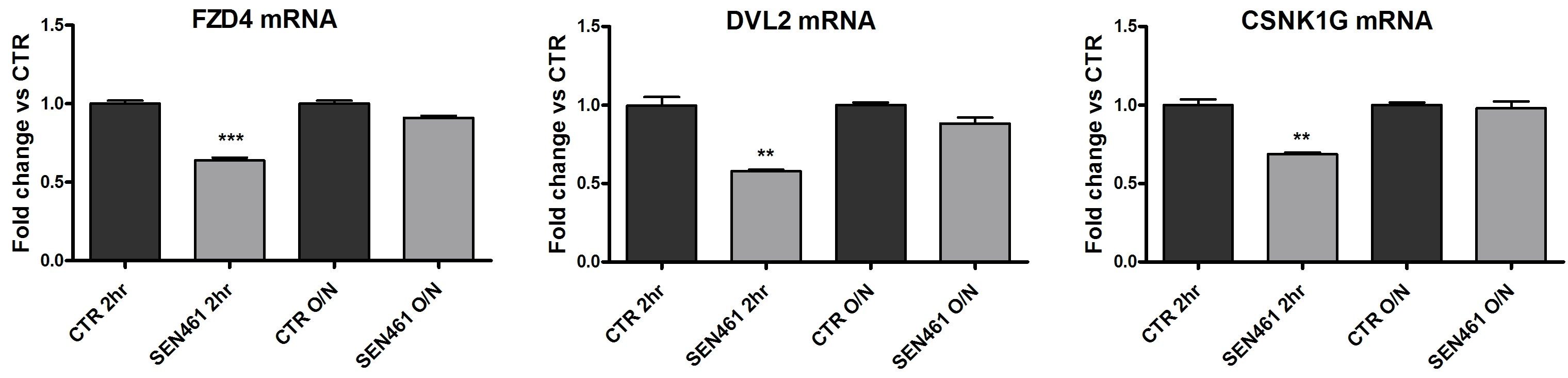

Supplement: Figure S3 — SEN461 effects on Wnt molecular components in U2OS cells. The effect of 10 µmol/L treatment with SEN461 on the mRNA levels of Wnt target genes FZD4, DVL2 and CSNK1G was measured by quantitative RT-PCR. Data represent means ± SEM. **, P<0.05 ***, P<0.005 (Student t test). (TIF) [file pone.0097847.s003.tif]

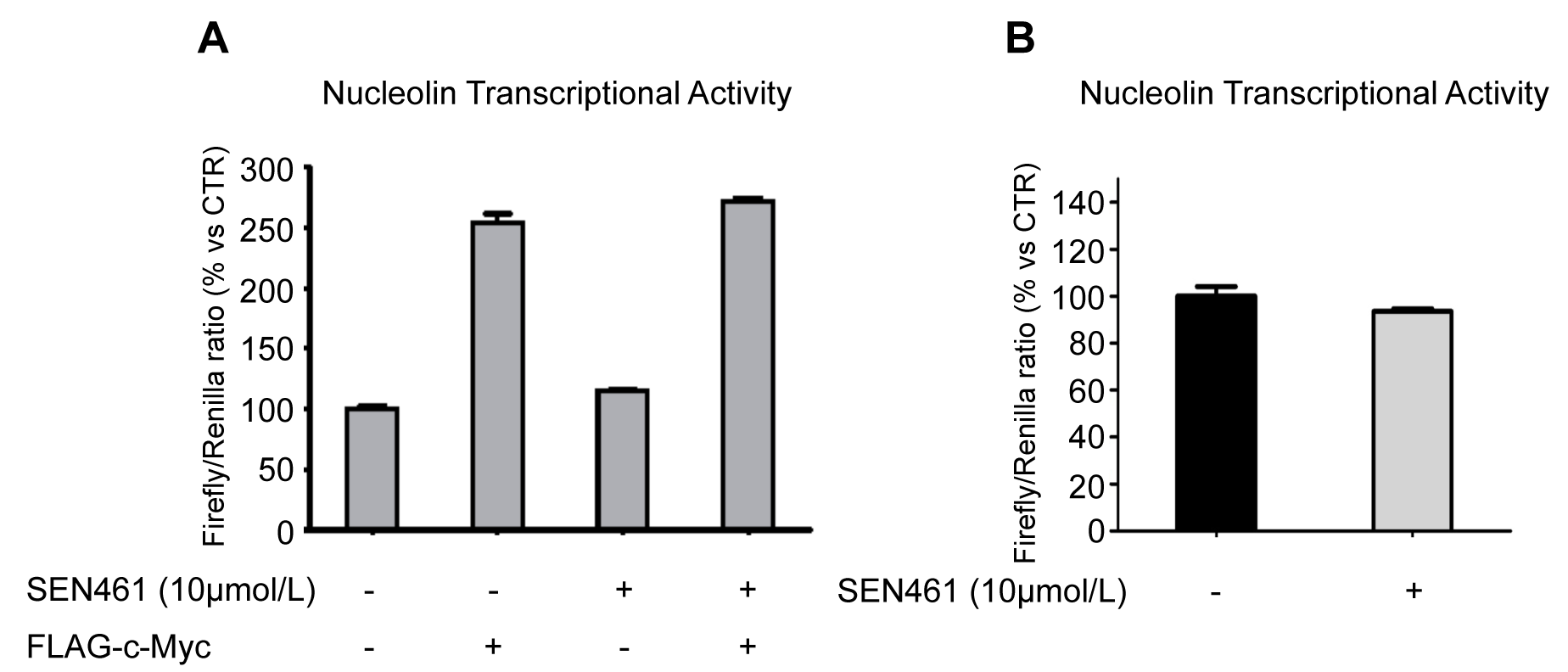

Supplement: Figure S4 — SEN461 doesn’t affect Myc transactivation of the nucleolin promoter. (A) HEK293 and (B) HT-1080 cells were transiently transfected with the mouse nucleolin reporter plasmid pNucL14 and the FLAG-c-Myc expression vector either alone or in combination and then treated with DMSO or SEN461. Data (from two independent experiments), normalized by cotransfection of TA-Renilla luciferase represent means ± SEM. (TIF) [file pone.0097847.s004.tif]

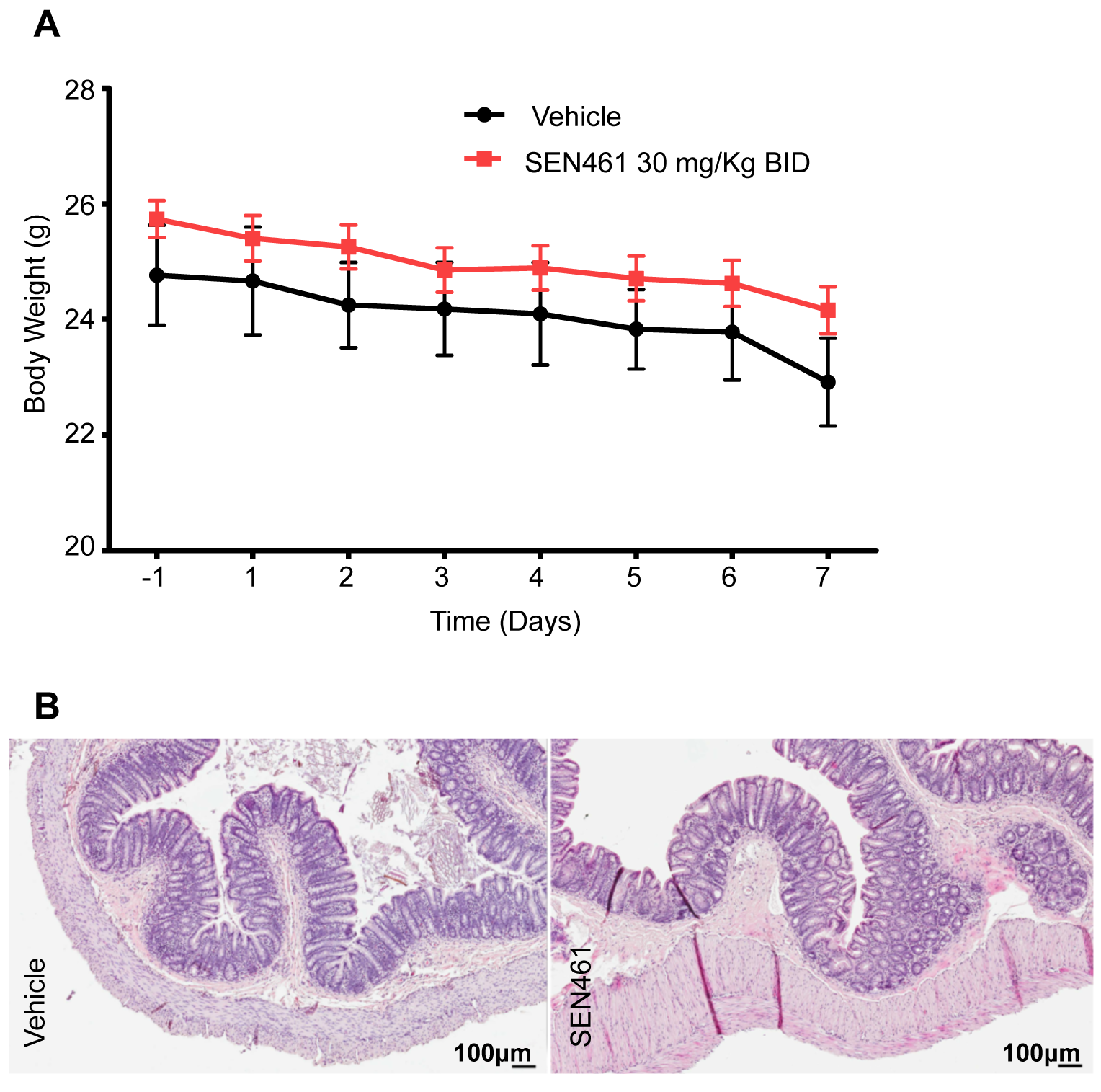

Supplement: Figure S5 — Effects of SEN461 on body weight and intestinal tissue. (A) Average body weight graph and representative histological sections (B) of intestinal tissue from mouse treated with vehicle or SEN461 for 7 days stained with hematoxylin and eosin. (TIF) [file pone.0097847.s005.tif]
